# THz Polarimetric Imaging of Carbon Fiber-Reinforced Composites Using the Portable Handled Spectral Reflection (PHASR) Scanner

**DOI:** 10.3390/s24237467

**Published:** 2024-11-22

**Authors:** Kuangyi Xu, Zachery B. Harris, Paul Vahey, M. Hassan Arbab

**Affiliations:** 1 Department of Biomedical Engineering, Stony Brook University, Stony Brook, NY 11794, USA; kuangyi.xu@stonybrook.edu (K.X.); zachery.harris@stonybrook.edu (Z.B.H.); 2 Boeing Research & Technology, Seattle, WA 98108, USA; paul.g.vahey@boeing.com

**Keywords:** polarimetry, terahertz imaging, non-destructive testing, carbon fiber panels, interwoven carbon fiber, polarimetric PHASR scanner

## Abstract

Recent advancements in novel fiber-coupled and portable terahertz (THz) spectroscopic imaging technology have accelerated applications in nondestructive testing (NDT). Although the polarization information of THz waves can play a critical role in material characterization, there are few demonstrations of polarization-resolved THz imaging as an NDT modality due to the deficiency of such polarimetric imaging devices. In this paper, we have inspected industrial carbon fiber composites using a portable and handheld imaging scanner in which the THz polarizations of two orthogonal channels are simultaneously captured by two photoconductive antennas. We observed significant polarimetric differences between the two-channel images of the same sample and the resulting THz Stokes vectors, which are attributed to the anisotropic conductivity of carbon fiber composites. Using both polarimetric channels, we can visualize the superficial and underlying interfaces of the first laminate. These results pave the way for the future applications of THz polarimetry to the assessment of coatings or surface quality on carbon fiber-reinforced substrates.

## 1. Introduction

Recently, terahertz (THz) technologies have rapidly advanced to achieve faster imaging speeds, compact and handheld form factors, and increased signal to noise ratios [1]. Thus, there is a renewed motivation to pursue compelling industrial applications of the THz technology. Due to its unique and distinct sensing capabilities, THz radiation is considered as a promising candidate for nondestructive testing (NDT) in industrial quality control [2]. Imaging with THz pulses (i.e., THz-TDS) can be formed similarly to how Pulse-Echo Ultrasonic Testing is typically applied; it also renders the cross-sectional or depth-resolved pictures of the specimen, which is crucial for evaluating samples of multi-layered structures [3]. This contributes to successful demonstrations made on composite materials, paints on car or airplane bodies, coatings of pharmaceutical tablets, art paintings, etc. [4]. Meanwhile, the implementations of THz technology have expanded beyond benchtop settings, for example, in clinical studies, or on the manufacturing lines of polymer, paper, or pharmaceutical industry [5,6].

Several comprehensive surveys on the NDT applications of THz technologies can be found in recent reviews [7,8,9,10]. Terahertz time-domain [11,12,13,14,15,16,17] and continuous wave spectroscopy [18,19,20,21,22] have been used in various NDT applications, in part due to the non-ionizing nature [23] and the sub-millimeter resolution of THz waves [24]. These applications include defect and delamination detection [25,26,27,28] as well as the measurement of coating thickness [29,30,31]. The accuracy of these measurements is highly dependent on the signal processing techniques employed (e.g., sparse deconvolution [30,32], stationary wavelet denoising [33,34], etc.) to extract the necessary quality control information.

Because most samples feature both intrinsic spatial variations and defect discontinuities, an imaging modality is usually preferred in NDT applications. While many choices of THz imaging systems are available, ranging from a point-scanning device to a single-shot camera, it is challenging to balance between competing performance measures, including the SNR, acquisition speed, resolution, field of view (FOV), and imaging contrast. Such optimizations can be seen in the recent development of our Portable Handheld Spectral Reflection (PHASR) scanners [35], a THz pulsed imaging device based on telecentric raster-scanning using an *f-θ* objective lens. So far, we have applied this device for the in vivo diagnosis of burn injuries [36,37,38], assessment of corneal edema [39,40], statistical analysis of speckle patterns [41], chemical imaging through scattering cloaks [42], etc.

In general, after a relatively complex signal processing process implemented on THz-TDS traces, the initial images of THz signals can be converted into maps of complex refractive indices. However, this procedure is less feasible due to poor SNR or limited knowledge of sample properties. Different processing methods are thus explored to interpret the spectroscopic information. Likewise, polarization-sensitive measurements are also incorporated to shed additional insights into sample structures. In this regard, the applications recognized by the existing THz research literature mainly involve (1) the visualization of surface properties (or roughness) [41,43], (2) intrinsic or stress-induced anisotropy of materials [44,45], (3) ellipsometry measurements on thin layers [46], (4) optical activity of biological structures [47,48], and (5) image enhancement [49]. Still, the progress towards the utilization of full polarimetric THz information in sample characterization has been limited. This is partially due to the deficiency of THz polarizing components, and the propagation of uncertainties when individual THz amplitude (intensity) signals are converted into polarimetric parameters [50]. To increase the speed, bandwidth, and accuracy of broadband THz polarization measurements, we have developed a spinning E-O sampling technique [51]. However, due to the need for image formation at high speeds in a portable form factor, using free-space 800 nm amplified laser beams is not practical. Therefore, to enable NDT applications, we recently developed a polarization-sensitive version of our PHASR scanner, enabled by two orthogonal PCA channels. The performance metrics of this device and corresponding calibration methods have been described elsewhere [52].

In this paper, we use the polarization-sensitive PHASR scanner for the imaging of carbon fiber-reinforced polymers (CFRPs) by mapping the THz Stokes vectors. In the NDT analysis of CFRP, the primary problem of interest lies in the evaluation of critical defect types, such as porosity, delamination, fiber defects, and low-velocity impact damage. Early investigations [25,53] had previously concluded that THz imaging “is generally not expected to be a major testing modality (for carbon materials) due to the conductivity of the material and therefore the rather limited penetration of terahertz into composites.” [54,55] While not optimistic about that “primary problem”, THz imaging can still serve to evaluate coatings on the carbon fiber substrate or characterize superficial properties. The most influential factor in this application of THz imaging is the anisotropic conductivity of CFRP, an intrinsic property that is also responsible for DC electrical or RF shielding testing [56,57]. It seems straightforward that currents along carbon fibers contribute to the reflection and screening of THz waves; however, the characterizations of these spectroscopic properties are limited and often inconsistent. For example, the maximum number of plies that can be resolved in independent THz studies have been significantly different, ranging from as many as eleven or as few as one [58,59,60,61]. Our experiments show that, in some CFRP materials provided by Boeing Company (Seattle, WA, USA), the penetration depth for the TE polarization of THz waves is limited to the first ply, whereas most of the TM polarization has been screened at the sample surface. The differences between the reflection spectra of TE and TM polarization are, thus, affected by the magnitude of surface echoes and the presence of subsurface echoes.

## 2. Materials and Methods

Our PHASR scanner incorporates the TERA ASOPS (Asynchronous Optical Sampling) dual-fiber-laser THz spectrometer (Menlo Systems, Inc., Newton, NJ, USA) into a handheld, collocated, telecentric imaging system. The detailed design, polarimetric calibration, and characterization of the performance metrics of the PHASR scanner have been previously reported [52]. Briefly, a THz beam generated by the photoconductive antenna (PCA) in the emitter is collimated using a TPX lens with a 50 mm focal length. The collimated beam is directed towards a gimballed mirror using a high-resistivity silicon beam splitter. The gimballed stage is a two-axis motorized system composed of a goniometer and a rotational stage, which raster-scans the collimated beam over the aperture of a custom-made telecentric *f-θ* lens. Therefore, the focused beam is always normally incident onto the target and has a constant focal spot size. A free-standing wire grid acts as a polarizing beam splitter, which separates the reflected radiation into the two orthogonal components denoted by X and Y. The signals from the two PCA detectors are converted by two transimpedance amplifiers, and then collected with two digital acquisition (DAQ) cards at synchronized times.

**Figure 1:**
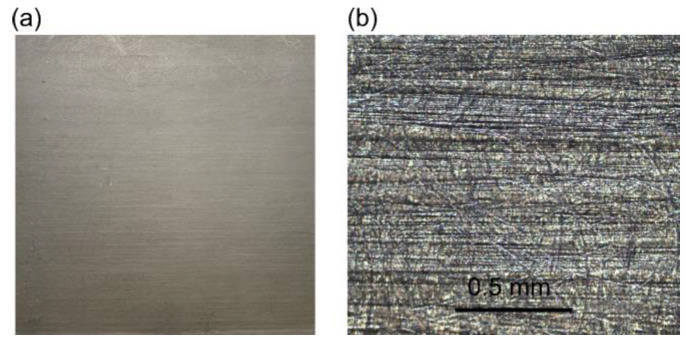
(**a**) Front surface of the first test panel from Boeing Company. (**b**) Microscopic image (10×) of the bare substrate, appearing as unidirectional CFRP.

**Figure 2:**
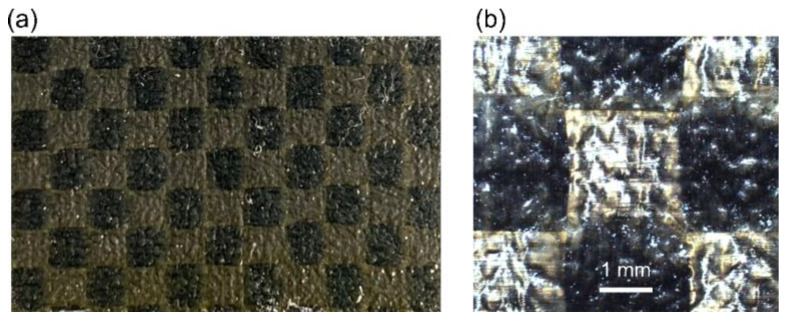
(**a**) Back surface of the second test panel from Boeing Company. (**b**) Microscopic image (2.5×) of the back surface, appearing as interwoven (plain-weaved) CFRP.

The CFRP testing panels were provided by Boeing Company and include two categories. The first panel is shown in Figure 1, which has multiple layers of coating on a CFRP substrate. The microscopic view of the uncoated substrate, shown in Figure 1b, shows that carbon fibers are aligned in the surface plane, i.e., the ply is unidirectional. The second panel is shown in Figure 2a, whose front surface is fully covered by coating, while the back surface is uncoated. Figure 2b is taken from an uncoated region of the back surface, which is investigated with THz imaging afterwards. This pattern of different “tiles” containing the orthogonal orientations of fibers is due to the plain weave, the dimensions of which are denoted as warp and weft. We will refer to the second class of targets as interwoven CFRP. When fibers aligned to a certain laboratory axis are assigned to a region of the sample, the linear polarization perpendicular to this axis is denoted as TE mode, and the one parallel to this axis is denoted as TM mode. Moving from the warp to the weft of interwoven CFRP, the directions of TE and TM modes will be switched.

These composites are placed at the focal plane of the PHASR scanner to form the THz polarimetric images. Each image pixel is recorded in 1 s by averaging 100 replicate traces in the time-domain. The size of FOV and pixel are optimized so that sufficient details of the sample profiles can be captured in reasonable imaging time. The polarization states of the THz source can be manipulated by rotating the PCA emitter and optionally adding a polarizing component. Finally, we have developed wavelet-domain signal analysis tools to investigate and mitigate any surface roughness present on the sample [62,63,64].

## 3. Results

### 3.1. Polarization-Sensitive Point Measurements

**Figure 3:**
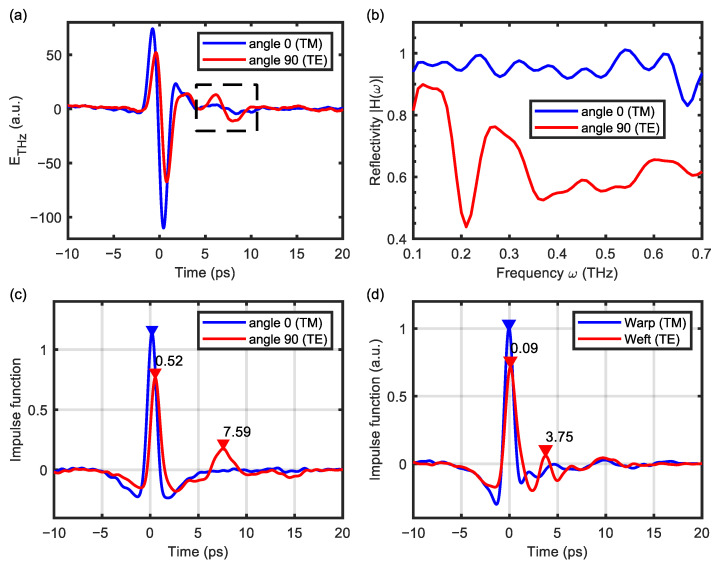
(**a**) The THz signals reflected from unidirectional CFRP at the sample orientations of 0° and 90°, corresponding to the TM and TE modes of polarization, respectively. The dashed box shows the difference in the propagation of the TE and TM modes in a single CFRP ply. (**b**,**c**) are the spectra of reflectivity and impulse responses retrieved from signals in (**a**). (**d**) The impulse responses are measured at different locations of interwoven CFRP, where the fiber orientations are different.

We start the signal processing with individual THz traces to determine the steps in data analysis. The unidirectional CFRP is a highly homogeneous ply and therefore, it is reasonable to compare the sample property as a function of the sample orientation angle. Figure 3a presents the reflected THz time-domain signals at the orientations of 0° and 90°, which is the angle between the fiber axis and the fixed linear polarization of the THz source. After deconvolving by a mirror reference measurement, the resulting spectra of the reflection are presented in Figure 3b. The reflection of the TE mode is overall weaker than that of the TM mode in either Figure 3a or Figure 3b, which is consistent with previous reports [53]. However, there are also exclusive dips below 0.4 THz in the spectra of the TE mode, which seem to be related to the “subsurface echoes” marked by the rectangle in Figure 3a. To substantiate this point, we can turn to the estimated impulse response of the sample, $\hat{h}(t)$, which is obtained from the following relations:

(1) $$w\left(t\right)=f_{HF}\;e\mathrm{xp}\left[-{\left(tf_{HF}\right)}^{2}\right]-f_{LF}\;e\mathrm{xp}\left[-{\left(tf_{LF}\right)}^{2}\right],$$

(2) $$\hat{h}\left(t\right)={\mathrm{FFT}}^{-1}\left\{H\left(\omega \right)\mathrm{FFT}\left[w\left(t\right)\right]\right\}.$$

where *H*(ω), the transfer function, is given by

(3) $$H\left(\omega \right)=\frac{\mathrm{FFT}\left[sample\left(t\right)\right]}{\mathrm{FFT}\left[reference\left(t\right)\right]},$$

and *w*(*t*) is a double Gaussian function given by the choice of $f_{HF}$ and $f_{LF}$, which have been set to 1.5 THz and 0.3 THz, respectively. This filter was first designed for analyzing the time-domain signals of tissue [65], and has been applied to CFRP later [60]. As shown in Figure 3c, two separate pulses can be recognized in the impulse response of unidirectional CFRP, with a relative time-delay of ∆*t* = 7.07 ps. Due to the destructive interference of these two pulses, spectral dips are expected to occur at 0.21 THz and 0.35 THz, which agrees well with the TE mode results in Figure 3b. This pattern of thin-film interference reveals the penetration depth of the THz waves in the testing panel. The TE mode can effectively propagate in the first ply of the unidirectional CFRP and be reflected at the next boundary, whereas the TM mode is significantly screened. For reference, the geometric thickness (*d*) of a single ply in this sample coupon was around 0.2 mm, while its optical thickness given by ∆*t* is 1.06 mm, equal to *d* multiplied by the THz refractive index (which we estimate between 4.4 and 5 [59]).

Next, we apply the same procedure to the measurements of interwoven CFRP, whose impulse responses are presented in Figure 3d. To address the spatial variation, imaging scans of 39 × 39 pixels are conducted on a stationary sample with an interval of 0.25 mm, while the THz emitter is set at an intermediate angle so that the reflections of two orthogonal polarizations are observable simultaneously. The blue and red curves in Figure 3d are captured at different pixels in the same polarimetric image (i.e., in the same detection channel), representing the THz signals of the warp and weft, respectively. Similarly, two separate peaks can be recognized in the TE mode (i.e., the weft); However, the relative time-delay is 3.66 ps, reduced by half compared to the unidirectional sample. It is likely that the TE mode is reflected at the interface between warp and weft within the first ply, and thus, the propagated distance is half of the ply thickness.

### 3.2. Polarization-Sensitive Imaging

**Figure 4:**
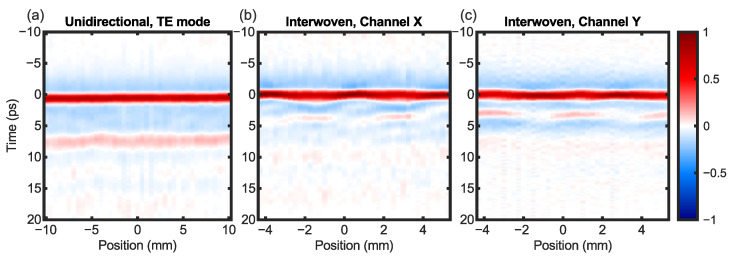
Cross-section images (B-scan) of (**a**) the unidirectional CFRP and the interwoven CFRP in the (**b**) X and (**c**) Y channels. The colors are on the same scale and have been extended to [−1, 1].

A complete set of data includes one pair of polarimetric images from the CFRP sample and another pair obtained using a reference mirror. Thus, the earlier signal processing steps are performed in each pixel. For comparison, we also conducted imaging scans on the unidirectional sample using 41 × 41 pixels with an interval of 0.5 mm. Figure 4 shows the cross-section view of the two CFRP samples, similar to the ultrasonic B-scan. In other words, each column of the image is the impulse response in one pixel, and the entire image constitutes a line scan. Two boundaries can be recognized in these figures: one is around *t* = 0, corresponding to the air–CFRP interface; the other corresponds to the subsurface echoes reflected by the interfaces in the first ply. In Figure 4b,c, the second echoes are more visible in the columns with weaker first echoes, offering an intuitive depiction of the screening effect of carbon fibers on THz E-field. The spatial pattern in the second echoes is reversed when comparing the two polarimetric channels, suggesting that the THz property of CFRP is well described by assuming “binary” responses to the TM and TE modes.

**Figure 5:**
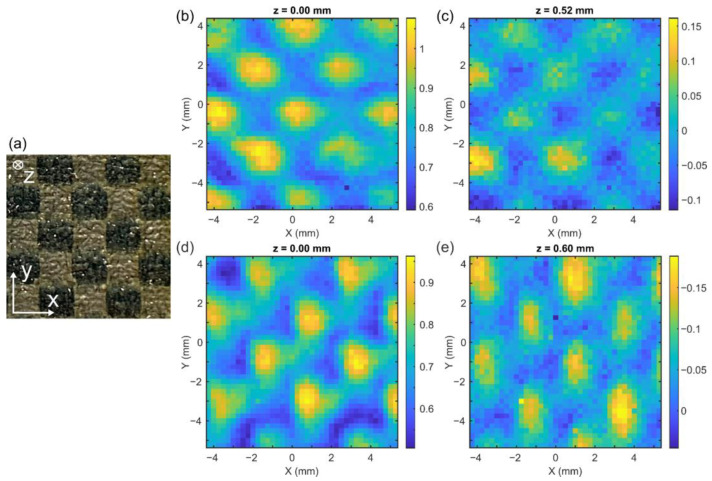
(**a**) Photo of the interwoven CFRP, top view. (**b**,**c**) are the C-scanned THz images of the X channel, at the optical depths of z = 0 and z = 0.52 mm, respectively. (**d**,**e**) are the correlated images of the Y channel, at the optical depths of z = 0 and z = 0.60 mm, respectively.

Alternatively, we can visualize the slices in the vicinity of the first and second boundary, resembling the ultrasonic C-scan. Figure 5 shows these *en face* THz images in comparison to the digital photo of a similar sample area. Despite the poorer spatial resolution, the THz images still render the profile of CFRP with abundant details. The excellent complementary relation between the two depth-resolved images from the x and y channels in the left and right column, Figure 5b,c, suggests the binary effect of warp and weft. Also, it can be seen that the spatial variation is related to the degree of screening.

**Figure 6:**
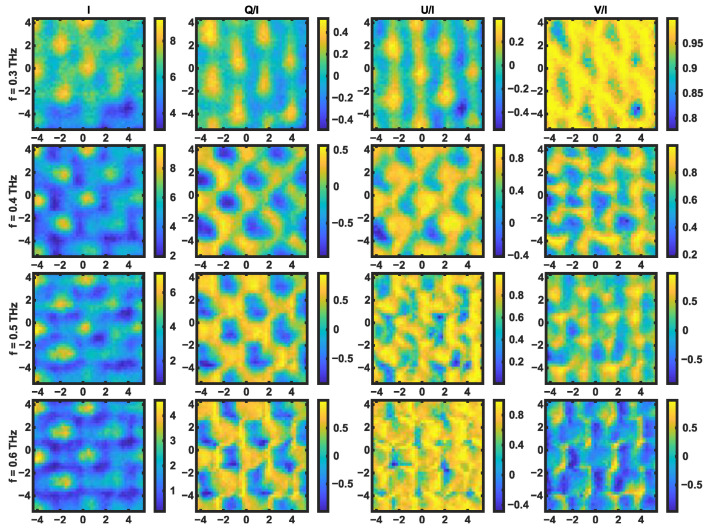
The spatial variation in the Stokes parameters *I*, *Q*, *U*, and *V* for the interwoven CFRP at different frequencies. *I* is in arbitrary units while the other Stokes parameters are normalized by *I*.

Lastly, we also investigated the joint functions of the two channels, such as the Stokes parameters. Although interesting textures have been observed from these polarimetric contrasts, as shown in Figure 6, it remains challenging to make objective interpretations. Also, according to our discussion on the thin-film interference, any spectral image below 0.4 THz is contributed by both echoes, resembling a blended picture of Figure 5b,c. Therefore, depending on the objectives of the NDT application, signal processing steps may be used to either employ or mitigate the second echo in this specific analysis. At any rate, Figure 6 clearly shows that the complementary regions of the sample are highlighted using the first two elements of the Stokes vectors (i.e., I and Q). Moreover, while the centers of the carbon fiber tiles are shown by the two intensity elements, the edges of the weave patterns are clearly resolved by the other two elements of the Stokes vector incorporating the phase information (i.e., U and V).

## 4. Discussion

**Figure 7:**
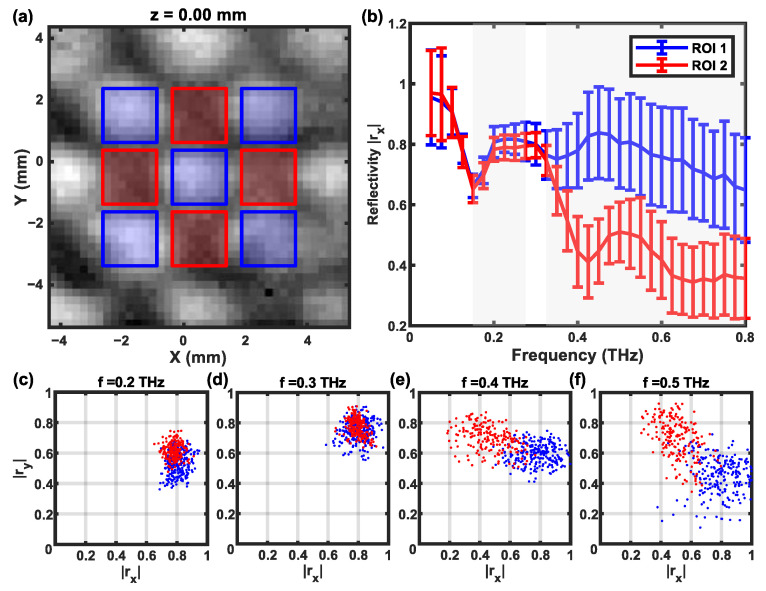
(**a**) Two ROIs (blue and red) are selected in the C-scan images of interwoven CFRP. (**b**) The mean value and standard deviation of the reflectivity in the two ROIs. (**c**–**f**) The distribution of pixels in the 2D plane of |R_x_| and |R_y_|, indicating the separation of orthogonal fibers in the 0.4–0.6 THz range.

Our experimental data indicate that the spectral range between 0.4 and 0.6 THz may be ideally suited to investigating carbon fiber samples. However, this ideal spectral range can be heavily affected by the thickness of the carbon fibers. To illustrate this point, Figure 7 shows the sensitivity of THz measurements in the identification of the fiber orientations.

Figure 7b shows the mean and standard deviations of the reflectivity over two ROIs associated with the warp and weft regions, defined in Figure 7a. The frequency ranges where ROI 1 and ROI 2 are statistically different (*p* < 0.01) have been marked with a gray shade. The contrast between the warp and the weft is more profound in the high-frequency range (>0.4 THz). On the contrary, the contrast at around 0.3 THz is not significant, which can be related to the thin-film interference that occurred in ROI 2. The sensitivity of classification can be seen intuitively in the 2D plane of Rx and Ry, shown in Figure 7c–f, using the reflectivity data of the same pixel in the X and Y channels, respectively.

The polarimetric results presented in this paper can be used to investigate the uniformity and intactness of carbon fiber samples through top coatings that are otherwise opaque to other wavelengths of light. These results also highlight the importance of considering the orientation of the sample and polarization-sensitive imaging using THz ellipsometry or polarimetry instruments. Understanding the strong polarimetric signatures of carbon fiber substrates in various structural forms can influence the interpretation of the recorded data. For instance, if the thickness of various coating layers is to be measured, the polarization of the incident THz beam and the orientation of carbon fibers should be considered.

## 5. Conclusions

We have performed polarization-sensitive THz testing on industrial carbon fiber composites using our PHASR imaging scanner. The spectroscopic properties of these samples are well described by a linear anisotropic model, represented by the axes of TM and TE polarizations. For either the unidirectional or interwoven CFRP material samples, the reflected signals of the TM mode only contain a single pulse, whereas those of the TE mode have a secondary pulse, which is a subsurface echo due to the first ply. The THz spectra of TM and TE polarizations can, thus, provide complementary information about the CFRP, as we have shown by mapping the Stokes vector of the reflected THz beam. This intrinsic anisotropy of CFRP can potentially lead to the application of THz polarimetric imaging in the assessment of coatings or inspection of other issues such as surface quality, fiber intactness, and uniformity.

## Data Availability

The data presented in this study are available upon request from the corresponding author.
